# Swallowing Function in COVID-19 Patients After Invasive Mechanical Ventilation

**DOI:** 10.1016/j.arrct.2021.100177

**Published:** 2022-01-11

**Authors:** Margareta Gonzalez Lindh, Gustav Mattsson, Hirsh Koyi, Monica Blom Johansson, Robin Razmi, Andreas Palm

**Affiliations:** a Department of Neuroscience, Speech and Language Pathology, Uppsala University, Uppsala, Sweden; b Centre for Research and Development (CFUG), Uppsala University, Region Gävleborg, Gävle, Sweden; c Department of Oncology-Pathology, Karolinska Biomics Center, Karolinska Institute, Stockholm, Sweden; d Section of Infectious Diseases, Department of Medical Sciences, Uppsala University, Uppsala, Sweden; e Department of Medical Sciences, Respiratory, Allergy and Sleep Research, Uppsala University, Uppsala, Sweden

**Keywords:** COVID-19, Critical care, Deglutition disorders, Frailty, Rehabilitation, Respiration, artificial, BMI, body mass index, BSE, bedside swallowing evaluation, CFS, Clinical Frailty Score, FOIS, Functional Oral Intake Scale, ICU, intensive care unit, IQR, interquartile range, SLP, speech and language pathology

## Abstract

**Objective:**

To explore swallowing function and risk factors associated with delayed recovery of swallowing in patients with COVID-19 post–invasive mechanical ventilation using the Functional Oral Intake Scale (FOIS).

**Design:**

Longitudinal cohort study.

**Setting:**

Three secondary-level hospitals.

**Participants:**

Invasively ventilated patients (N=28) who were hospitalized with severe COVID-19 and referred to the hospitals’ speech and language pathology (SLP) departments after mechanical ventilation between March 5 and July 5, 2020 for an evaluation of swallowing function before commencing oral diet.

**Interventions:**

SLP assessment, advice, and therapy for dysphagia.

**Main Outcome Measures:**

Oral intake levels at baseline and hospital discharge according to the FOIS. Patients were stratified according to FOIS (1-5, dysphagia; 6-7, functional oral intake). Data regarding comorbidities, frailty, intubation and tracheostomy, proning, and SLP evaluation were collected.

**Results:**

Dysphagia was found in 71% of the patients at baseline (79% men; age, 61±12y; body mass index, 30±8 kg/m^2^). The median FOIS score at baseline was 2 (interquartile range [IQR], 1) vs 5 (IQR, 2.5) at hospital discharge. Patients with dysphagia were older (64±8.5y vs 53±16y; *P*=.019), had a higher incidence of hypertension (70% vs 12%; *P*=.006), and were ventilated invasively longer (16±7d vs 10±2d; *P*=.017) or had a tracheostomy (9±9d vs 1±2d; *P*=.03) longer. A negative association was found between swallowing dysfunction at bedside and days hospitalized (*r*=–0.471, *P*=.01), and number of days in the intensive care unit (ICU) (*r*=–0.48, *P*=.01).

**Conclusion:**

Dysphagia is prevalent in COVID-19 patients after invasive mechanical ventilation and is associated with number of days in hospital and number of days in the ICU. Swallowing function and tolerance of oral diet improved at discharge (*P*<.001).

During the first surge of the COVID-19 pandemic, between 7% and 8% of patients hospitalized with COVID-19 were admitted to the intensive care unit (ICU).^1^ The primary reason for hospitalization was respiratory failure. Dysphagia (swallowing dysfunction) is prevalent after prolonged mechanical ventilation (>48h).^2^ Invasive ventilation can have a negative effect on laryngeal competence and swallowing physiology^2^^,^^3^ due to edema, vocal fold immobility, reduced sensation, and muscle disuse.^4^ Time intubated is the strongest risk factor for dysphagia after invasive mechanical ventilation, with the incidence rate varying depending on the cohort studied and how dysphagia is defined.

A systematic review by Skoretz et al^5^ of 14 studies with a total of 3520 patients (medical, surgical, and cardiovascular surgical) after endotracheal intubation found a reported dysphagia frequency ranging from 3% to 62%, where the highest dysphagia frequencies included patients experiencing prolonged intubation (>24h). More than half of the included studies reported a dysphagia frequency exceeding 20% and dysphagia associated with pneumonia, prolonged treatment of antimicrobial therapy, reintubation, tracheostomy, prolonged hospital and ICU length of stay, and increased short- and long-term mortality.

Brodsky et al^6^ followed acute respiratory distress syndrome survivors (n=37) with symptoms of dysphagia after oral intubation prospectively over a 5-year period postdischarge. They found that the median time to recovery was 3 months (interquartile range [IQR], 3-6) with 23% of survivors having symptoms persisting more than 6 months. All resolved within 5 years after hospital discharge.

Prone positioning has been found to reduce mortality among patients with moderate-to-severe acute respiratory distress syndrome^7^ and has become the standard of care for COVID-19 patients. There are presently no data on whether or not prone positioning affects swallowing function after mechanical ventilation in general, or whether patients with COVID-19 are particularly vulnerable due to their frequent need for prolonged ICU stays.

Dysphagia assessment and treatment are generally performed by a specialist, often a speech and language pathology (SLP) specialist, but can also be performed by other professions (eg, phoniatricians, otolaryngologists, occupational therapists, or critical care physicians).^4^ An instrumental evaluation is often recommended as a complement to a clinical bedside examination,^8^ with either a flexible endoscopic evaluation of swallowing or videofluoroscopy (also called modified barium swallow). However, both methods are considered aerosol-generating procedures and were restricted during the COVID-19 pandemic.^9^

Dysphagia has been identified as one of the most important sequelae of severe and critical forms of COVID-19.^10^ However, the magnitude of short- and long-term dysphagia in patients with COVID-19 is not yet known.

The aims of this study were threefold: to determine the incidence and grade of dysphagia in patients with COVID-19 after mechanical ventilation using level of oral intake, to determine recovery rate, and to explore risk factors associated with dysphagia. In this article, the terms dysphagia and swallowing dysfunction will be used synonymously.

## Methods

### Participants

This was a longitudinal cohort study of consecutive patients aged 18 years and older with a positive real-time reverse-transcriptase polymerase chain reaction test for SARS-CoV-2 admitted to 3 ICUs in the region (285,452 inhabitants). Patients who contracted COVID-19 while already in the hospital were excluded. Patients were referred to the hospitals’ SLP departments after mechanical ventilation between March 5 and July 5, 2020 (service 5d/wk) for an evaluation of swallowing function before commencing oral diet. This is a substudy of the Gävleborg COVID-19 cohort study. Data regarding age; clinical frailty evaluated with the Clinical Frailty Score (CFS)^11^; smoking, respiratory, and swallowing parameters; comorbidities; days with a tracheostomy; total days with a ventilator; total days of hospitalization; prone position; and days between extubation/decannulation and bedside swallowing evaluation were recorded. Body mass index (BMI) was calculated from body weight in kilograms divided by height in meters squared (self-reported or from medical chart).

### Setting

Patients were evaluated by an SLP either in the ICU, high dependency unit, or in the COVID-19 ward after being transferred from the ICU. Recommendations were subsequently given regarding oral intake of medication, liquids, and food. The patients were monitored until return of safe oral feeding or until discharged to a rehabilitation clinic.

### Bedside swallowing evaluation

A bedside swallowing evaluation (BSE) was performed when the patients were deemed medically stable and awake after mechanical ventilation. It was performed with the patient in an upright position to assess motor (strength, speed, and range of movement) and sensory function of intraoral musculature, cranial nerve examination, respiratory function, ability to follow single-step verbal commands, dentition, cough quality, and dysphonia. Pulse oximetry was performed, and oxygen support and respiratory rate was recorded. The patients were observed swallowing different liquids, consistencies, and volumes using the Volume Viscosity Swallowing Test,^12^ but adding a solid bolus (typically a dry cracker) and a larger volume of water (100 mL) when appropriate.^13^ Clinical signs of impaired safety of swallowing (cough, decrease in oxygen saturation, or change in voice quality) and impaired efficacy (bolus retention, posterior bolus leak, multiple reswallows, and difficulty initiating a swallow), were analyzed, as well as laryngeal palpation when possible. Oral intake recommendations were based on a patient's swallowing ability in combination with other factors such as delirium, postural control, and fatigue.

### Functional Oral Intake Scale

The Functional Oral Intake Scale (FOIS)^14^ is the most frequently used scale for evaluation of oral intake and was used as an outcome measure of swallow function. The FOIS is a validated 7-point ordinal scale: 1 (nothing by mouth), 2 to 3 (tube dependent), 4 (total oral intake of a single consistency), 5 (total oral intake of multiple consistencies requiring special preparation), 6 (total oral intake with no special preparations, but minimal restrictions), and 7 (total oral diet with no restrictions). Patients were stratified according to swallowing function, where FOIS level 1 to 5 was defined as dysphagia and level 6 to 7 as functional swallowing. The oral intake recommendation at hospital discharge was used to determine the secondary outcome measure.

### Follow-up

All patients were invited to answer the 4-point swallowing questionnaire test^15^ 1 to 2 months after discharge from the hospital or rehabilitation clinic.

### Ethical considerations

This study was approved by the Swedish Ethical Authority (Dnr 2020-01746). Informed consent was obtained from all patients.

### Statistical analysis

Normally distributed continuous data were presented as mean ± standard deviation and non-normally distributed data as median with IQR. Categorical data were presented as frequencies and percentage. The difference between groups was analyzed with the Student *t* test for normally distributed continuous data, with the Mann Whitney *U* test for non-normally distributed continuous data, and the chi-square test for categorical variables. The association between FOIS at ICU discharge (baseline) and number of days in the hospital, number of days in the ICU, age, BMI, number of days intubated, prone position, frailty, and tracheostomy were analyzed using Spearman's rank correlation coefficient. A P value of less than .05 was regarded as significant. Statistical analyses were conducted using Stata, version 16.1.^a^

## Results

In total, 28 patients were included in the study (79% men; age, 61±12y; age range, 25-78y; BMI, 30±8 kg/m^2^). Baseline characteristics are presented in table 1. All patients lived at home and had a median clinical frailty score of 3 (range, 1-5) before hospitalization with COVID-19. No patients had previous dysphagia or neurological diseases. Prone position was applied in 16 of 28 patients (57%); however, the length of time prone could not be determined from the medical records. The median length of ICU stay was 20 days (IQR, 17-31d) and the median hospital stay was 35 days (IQR, 27-52d). Delirium was evident in 61% of the patients at BSE. One patient died during hospitalization. Of the surviving 27 patients, 41% (n=11) were discharged home and the others to specialized rehabilitation clinics.

**Table 1:** Demographic and clinical characteristics stratified according to swallowing function at BSE

Characteristics	Total	Functional Swallow (FOIS 6-7)	Dysphagia (FOIS 1-5)	*P* Value
	N=28	n=8	n=20	
Age, mean (SD), years	61.0 (11.9)	52.9 (15.6)	64.2 (8.5)	.019*
Men, n (%)	22 (79)	7 (88)	15 (75)	.47
BMI, mean (SD), kg/m^2^	30.1 (7.9)	30.7 (10.8)	29.9 (6.7)	.79
CFS, n (%)				.44
1	1 (4)	1 (12)	0 (0)	
2	6 (21)	2 (25)	4 (20)	
3	18 (64)	4 (50)	14 (70)	
4	2 (7)	1 (12)	1 (5)	
5	1 (4)	0 (0)	1 (5)	
Smoking status, n (%)				
Ex-smoker	35 (9)	3 (38)	6 (3)	
Never smoker	58 (15)	5 (62)	10 (5)	
Smoker	8 (2)	0 (0)	2 (1)	
Prone position, n (%)	16 (57)	3 (38)	13 (65)	.18
Chronic cardiac disease, n (%)	4 (14)	0 (0)	4 (20)	.17
Hypertension, n (%)	15 (54)	1 (12)	14 (70)	.006*
Diabetes, n (%)	6 (21)	1 (12)	5 (25)	.47
Duration of orotracheal intubation, mean (SD)	14.1 (6.5)	9.6 (2.1)	15.9 (6.8)	.017*
Days in ICU, median (IQR)	20.0 (14.5)	15.5 (4.5)	28.5 (18.5)	<.001*
Tracheostomy, n (%)	14 (50)	2 (25)	12 (60)	.094
Days with tracheostomy, mean (SD)	7 (8.6)	1.1 (2.2)	9.4 (9.1)	.03*
Days in hospital, median (IQR)	35.0 (25.3)	24.0 (10.3)	46.5 (24.3)	.003*
Days from extubation/decannulation to SLP evaluation, mean (SD)	3.4 (2.6)	4.6 (3.1)	3.0 (2.3)	.12
Discharged home, n (%)	11 (41)	8 (100)	3 (16)	<.001*
Discharged to rehabilitation, n (%)	16 (59)	0 (0)	16 (84)	<.001*
Diseased, n (%)	1 (5)	0 (0)	1 (8)	.42

⁎ Significant *P* values.

### Prevalence of dysphagia

Clinical signs of swallowing dysfunction (FOIS 1-5) were found in 20 of 28 patients (71%) (table 1); the median FOIS was 2 (IQR, 1). Complete or partial feeding tube dependency (FOIS 1-3) was seen in 57% of the patients (n=15). Three patients were assessed as FOIS 6, which means that some food or liquid items must be avoided. We chose to categorize these patients to the “functional swallowing group” because they were eating food from the regular hospital menu, although only the easy-to-chew options.

The main presenting dysphagia symptoms were oral and pharyngeal muscle weakness (71%), cough (50%), and bolus retention (32%) (table 2). Patients with dysphagia were older (64±8.5y vs 53±16y; *P*=.019), had a higher incidence of hypertension (70% vs 12%; *P*=.006), and remained with invasive ventilation (16±7d vs 10±2d; *P*=.017) or tracheostomy (9.4±9.1d vs 1.1±2.2d; *P*=.03) longer. The median length of ICU stay (28.5d [IQR, 18.5d] vs 15.5d [IQR, 4.5d], *P*=.001) and length of hospitalization (46.5d [IQR, 24.3d] vs 24.0d [IQR, 10.3d], *P*=.003) were longer.

**Table 2:** Respiratory vitals and swallowing symptoms at SLP evaluation

Parameters	Total	Functional Swallowing (FOIS 6-7)	Swallowing Dysfunction (FOIS 1-5)	*P* Value
	N=28	n=8	n=20	
**Respiratory vitals**				
Breaths per minute	22.6 (4.0)	20.4 (2.2)	23.6 (4.2)	.053
Oxygen saturation, % (SD)	91.8 (17.8)	95.4 (2.2)	90.2 (21.4)	.50
Oxygen by nasal cannula, n (%)	18 (64)	3 (11)	15 (54)	.64
High flow nasal cannula, n (%)	5 (18)	1 (12)	4 (20)	.64
**Swallowing and voice symptoms, n (%)**				
Posterior leak	7 (25)	1 (12)	6 (30)	.26
Bolus retention	9 (32)	3 (38)	6 (30)	.28
Multiple reswallows	6 (21)	3 (38)	3 (15)	.26
Oral muscle weakness	20 (71)	2 (25)	18 (90)	<.001
Weak mastication	8 (29)	3 (38)	5 (25)	<.001
Cough	14 (50)	1 (12)	13 (65)	.035
Wet voice	4 (14)	0 (0)	4 (20)	.17
Drop in oxygen saturation	2 (7)	1 (12)	1 (5)	.15
Pharyngeal muscle weakness	20 (71)	2 (25)	18 (90)	<.001
Fatigue	26 (93)	6 (75)	20 (100)	.020
Dysphonia bedside	27 (96)	8 (100)	19 (95)	.52

### Respiratory function after mechanical ventilation

Fifty percent of the patients (n=14) had been tracheotomized (table 1), but 11 of them were decannulated at the time of bedside evaluation. Reintubation occurred in 7 (25%) of the patients and 3 times in 1 patient. The mean length of time from tracheostomy insertion to decannulation was 7 days (SD, 8.6d). See table 2 for respiratory vitals at BSE.

### Recovery rate

**Fig 1:**
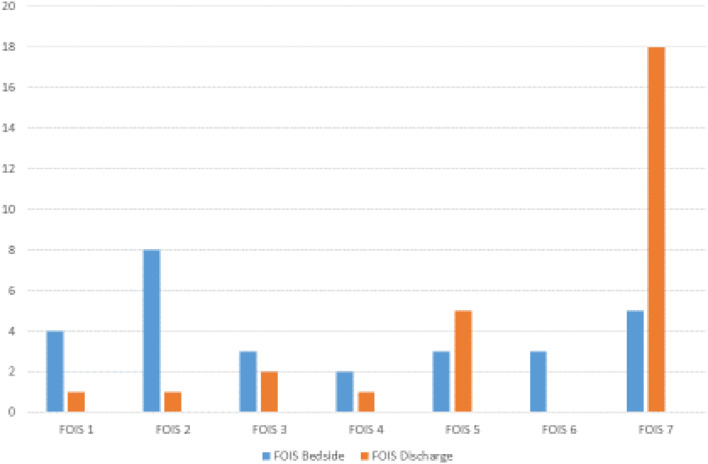
Number of patients with each FOIS score at BSE and hospital discharge.

At discharge from the hospital, all patients had been decannulated and 47% (n=9) of the patients with a FOIS of 1 to 5 at BSE had recovered a functional oral intake (FOIS 7). Of the 11 patients discharged home, 1 remained with restrictions in oral intake (FOIS 5). In the group discharged to the rehabilitation clinic, 56% (9 of 16) remained with diet restrictions (FOIS 1-5), with 4 patients (15%) having complete or partial tube dependency (FOIS 1-3). Figure 1 shows the distribution of FOIS score at BSE and hospital discharge.

### Follow-up

In total, 79% (n=22) of the patients attended a follow-up visit 8 weeks (IQR, 3.75) after discharge from the hospital. Of the 6 patients lost to follow-up, 1 patient cancelled the appointment. The remaining 5 were lost due to death (n=1), the patient returning to his home country (n=1), the patient living in another region (n=2), or the patient being followed at the local clinic (n=1). Dysphagia had resolved in 13 of the 14 patients (93%), with the remaining patient reporting mild dysphagia symptoms. One of the patients in the “no dysphagia group” at discharge reported mild dysphagia symptoms at follow-up. The reported symptoms were that “it takes longer to eat meals than it used to” and “swallowing is effortful.” Information on taste, smell, nutrition, and voice complaints are reported in table 3. If the patient had skipped a question and did not comment on it as being a problem in the conversation with the physician, it was scored as having no problem.

**Table 3:** Patient-reported symptoms at follow-up visit (n=22)

Symptom	None	Mild	Moderate
Dysgeusia (taste), n (%)	13 (59)	9 (41)	
Anosmia (smell), n (%)	14 (64)	8 (36)	
Nutritional problems, n (%) *	21 (96)	1 (4)	
Dysphonia, n (%)	10 (45)	10 (45)	2 (9)

⁎ Difficulty eating and drinking enough, weight loss.

### Associated risk factors

**Fig 2:**
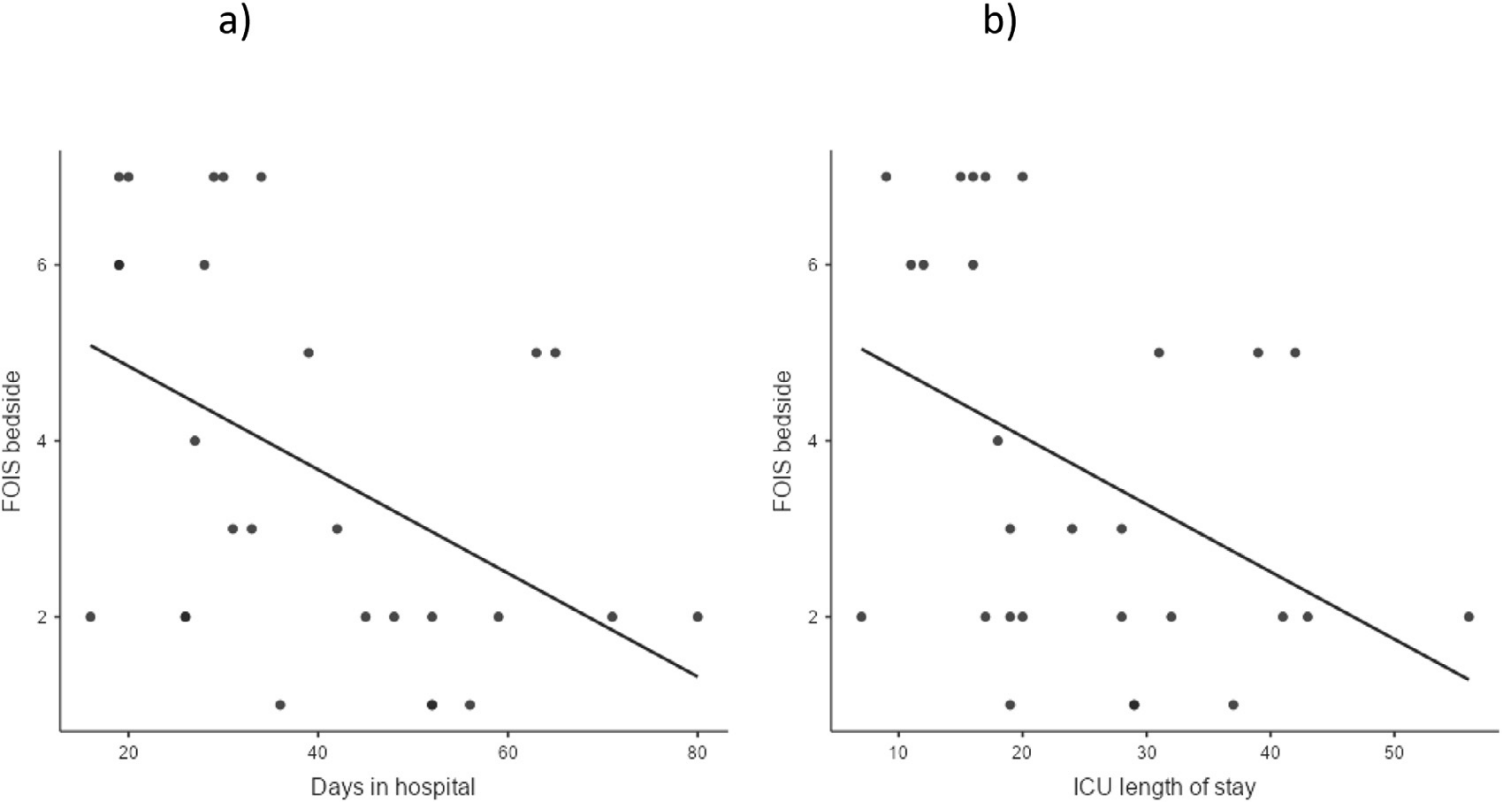
Scatterplot with regression line depicting the relationship between FOIS level and (A) number of days in the hospital and (B) number of days in the ICU.

A moderate negative association was found between swallowing function at BSE and number of days in hospital (*r*=–0.471, *P*=.01; fig 2A) and between number of days in the ICU (*r*=–0.48, *P*=.01; fig 2Bb), as well as needing nutritional support at discharge (*r*=–0.445, *P*=.02). There was a moderate association between FOIS at baseline and whether a patient was discharged home or to a rehabilitation clinic (*r=–*0.541, *P*=.004). No significant associations were found between FOIS level at baseline and age, BMI, number of days intubated, prone position, CFS, or having had a tracheostomy (*P*>.05).

## Discussion

This longitudinal cohort study found that dysphagia frequency after invasive mechanical ventilation in patients with COVID-19 was high, with 71% requiring significant nutritional and swallowing interventions. This is in accordance with emerging data on this patient group.^16^ Patients presented most frequently with signs of oral and pharyngeal muscle weakness at the BSE but also with significant fatigue and delirium, indicating that the dysphagia was multifactorial.

Despite the average length of intubation far exceeding the time known to increase the risk of swallowing dysfunction,^5^ there was a rapid trajectory of improvement with the majority of patients (85%) having a full oral intake on 1 or multiple consistencies at discharge from hospital to the rehabilitation clinic (fig 2). This is in accordance with results presented by Lima et al,^17^ in which 101 ICU patients diagnosed with COVID-19 were compared with 150 critical ICU patients with prolonged orotracheal intubation (≥48h) from the same institution. Dysphagia after prolonged intubation was common in both groups of their study. However, despite patients with COVID-19 remaining intubated longer than the other group, they had less sustained dysphagia at discharge.^17^ Dysphagia after mechanical ventilation can be multifactorial. It can be the direct result of the underlying problem requiring ICU admission (medical and/or surgical) but can also be acquired as a result of ICU care.^18^ Further studies on the underlying causes of variations in dysphagia resolution are needed.

Frailty was screened on admission using the CFS,^11^^,^^19^ which has been validated as a predictor of outcomes in older people. The CFS is now increasingly being used as a triage tool to make clinical decisions in the management of patients with COVID-19.^19^ A CFS score of 5 is the most widely used cutoff point to define frailty (fit [1-3], pre-frail [4-5], and frail [≥6]). In this cohort 25 of 28 patients were categorized as fit, which might partly explain the rapid improvement and that no association was found between swallowing dysfunction and age or number of days invasively ventilated.

In total, 15 patients (57.1%) were completely or in part feeding tube dependent (FOIS 1-3) at the BSE. This number had decreased to 4 patients (15%) at hospital discharge, and the remaining patients (n=11) were discharged on an oral diet without feeding tube dependency to either home or a specialized rehabilitation facility. This demonstrates a rapid and progressive improvement in the cohort but does not provide detailed information regarding swallowing physiology, as no instrumental evaluations were performed.

Emerging data suggests that prone positioning might not have the negative effect on swallowing that has been hypothesized.^20^ If and how it influenced swallowing function in this cohort cannot be established due to missing data in the medical charts.

Tracheostomy was performed in 50% of the patients. There was a good success of weaning, with the majority decannulated before BSE and all patients decannulated at discharge. This is in accordance with the case series presented by Cardasis et al,^21^ in which 74% of their 24 patients were decannulated at discharge from hospital. Like theirs, our cohort had a high baseline level of health, with a median Clinical Frailty Score of 3 pre-COVID-19.

Although dysphagia was common at bedside evaluation, the prognosis for resolution of dysphagic concerns seems good and recovery of swallowing function in patients with COVID-19 after invasive mechanical ventilation was high. Only 2 patients reported some element of dysphagia at follow-up. In contrast, 54% (n=12) reported dysphonia and were referred for SLP evaluation. This is consistent with emerging data from other countries.^17^^,^^20^

The factors most strongly associated with dysphagia in this cohort, prolonged hospital length of stay and ICU length of stay, did not differ from those identified in the review by Skoretz et al^5^ and Brodsky et al.^18^ However, restrictions in oral intake seemed to resolve faster in this group of COVID-19 patients.

### Study limitations

The strengths of this study were the longitudinal design and that patient-related outcome measures (4-point swallowing questionnaire test) were collected at follow-up, which is valuable when determining patients’ perception of their outcome. However, the study also had several limitations, including a small sample size and that only patients referred to SLP were included. Swallowing function was only measured by FOIS and, although this is a validated method of estimating the functional eating ability of a patient, it does not analyze the biomechanical aspects of swallowing, which is important when designing interventions for improving swallowing function. It also does not take patients’ subjective perception of swallowing into consideration. However, oral intake is probably a more patient-centered and meaningful outcome compared with physiological swallow measures from the patient perspective, as argued by Regan et al.^22^ When using a clinical judgment in (any) assessment of an impairment, there is always a risk of bias. In this study, we used validated scales such as FOIS and the clinical frailty scale in an effort to control for inter-rater bias. Finally, follow-up data were based on patient-reported outcome measures, not a clinician-rated scale, which means that there were some inconsistencies in how swallowing symptoms were expressed.

## Conclusions

In this study, the majority of patients with COVID-19 needed precautionary measures to ascertain a safe oral intake after mechanical ventilation. We therefore recommend that screening of swallowing function be added to local ICU policies. In circumstances such as these, where the aerosol-generating aspects are uncertain, the best practice for assessing swallowing function in COVID-19 patients is a carefully executed BSE, to avoid further potential stressors on a reduced lung function.

### Significance

The results of this study provide new knowledge regarding prevalence, assessment, and outcome for this new patient group, both to medicine in general and to speech pathology in Sweden. We have also gained new knowledge about factors associated with swallowing dysfunction.

## Supplier

a. Stata, version 16.1; StataCorp LP.
